# Identification of the factors determining the concentration and spatial distribution of Zn, Pb and Cd in the soils of the non-forest Tatra Mountains (southern Poland)

**DOI:** 10.1007/s10653-022-01201-3

**Published:** 2022-01-10

**Authors:** Krystyna Ciarkowska, Anna Miechówka

**Affiliations:** Soil Science and Agrophysics Department, University of Agriculture, Aleja Mickiewicza 21, 31-120 Krakow, Poland

**Keywords:** Trace metal accumulation, Carbonate-bearing soils, Carbonate-free soils, Ecological risks, Vegetation belt, Slope, Exposure

## Abstract

We investigated trace-metal (TM)––Zn, Pb and Cd––concentrations and spatial distributions in the uppermost layers of non-forest soils from Tatra National Park (West Carpathians). We aimed to determine the main factors affecting the distribution of TMs, as well as the risk they posed to the environment. TM concentrations were compared to the target and intervention values established by the Dutch Ministry. Principle component analysis was used to identify the potential factors affecting TM accumulation, with two-factor analysis being applied to further examine the importance of any given factor. To examine the regularity of the TM distribution, semivariograms were created. The semivariograms of Cd and Pb were similar, suggesting a moderate spatial dependence for these metal concentrations, while the Zn variogram indicated a lack of spatial continuity for this metal. We established that the Zn, Pb and Cd exceeded target levels and at some sites, Cd exceeded the intervention values, posing a strong ecological risk to the environment. Our study confirmed that the parent rock was the most important factor affecting the TM accumulation. The carbonate-free soils differed from carbonate soils in the second important factor affecting TM accumulation, for carbonate-free soils it was location when for carbonate soils–TM content in the parent material. The Zn, Pb and Cd distribution patterns indicated that Cd, but also to a lesser degree Pb and Zn, accumulation mainly resulted from long-range transport from industrialised areas, while the Zn concentrations were also affected by local sources, such as the historical mining of Zn ore.

**Supplementary Information:**

The online version contains supplementary material available at 10.1007/s10653-022-01201-3.

## Introduction

The trace metals (TMs) Zn, Pb and Cd are the most widespread in the environment, abundant not only in industrialised and urban regions but also in remote areas (Jaguś & Skrzypiec, 2019; Szopka et al., 2013; Tomaškin et al., 2013). The natural concentrations of TMs in soils vary primarily as a function of the mineral composition of the parent rock, its chemical alteration and pedogenesis, which result in the formation of the soil properties (Barančoková et al., 2009; Kowalska et al., 2021; Santos-Francés et al., 2017; Utermann et al., 2019). The TM concentrations can be diverse, depending on the rock origin and type, while soil organic-matter content, pH, and sorption capacity are among the most important soil properties that influence TM accumulation (Barančoková et al., 2009; de Vries et al., 2007; Hudec et al., 2013).

An important source of TMs in soils is atmospheric pollution resulting from anthropogenic activities, such as emissions from the industrial and automotive sectors. Because TMs can travel long distances from their point of emission, they can be deposited a long way away from the source (Briffa et al., 2020). The effects of TM deposition are particularly well expressed in mountainous areas because mountain ranges are characterised by a much higher volume of precipitation than lowlands and play the role of orographic barriers to moving masses of air (Smidt & Herman, 2004; Szopka et al., 2013). As a consequence, the long-range atmospheric transport of TMs has resulted in their deposition even in areas that are supposed to be pristine, such as national parks (Grodzińska et al., 1990; Mazurek et al., 2017; Tomaškin et al., 2013).

The Tatra Mountains, part of the West Carpathians, form the border between Poland and Slovakia. A minor portion of their area in Poland has national park status. The location of Tatra National Park (TNP) means this area is subject to significant amounts of pollution coming from the industrialised areas of southern Poland and neighbouring countries (Ciriaková, 2009; Miechówka et al., 2002; Wieczorek & Zadrożny, 2013). In addition to long-term emissions, local and traffic emissions also contribute significantly to the pollution (Paukszto & Mirosławski, 2019). Zakopane, a small town of around 28,000 inhabitants, is situated at the foot of the Tatra Mountains and provides tourism services throughout the year, being frequented by more than two million tourists annually. Therefore, there is heavy traffic throughout the year, with the large amount of tourists requiring accommodation, which results in the additional production of a significant amount of pollutants (Ciarkowska, 2018).

Natural and anthropogenic sources of TMs are often superimposed on the soil and it is very difficult to separate the contributions from these sources. Thus, statistical methods, such as principal component analysis (PCA) and multivariate analyses are used, which can identify a pollution source by analysing the metal associations in each principal component (Hu et al., 2013; Wei et al., 2010; Zhang et al., 2009). Due to natural variability and widespread and diffuse anthropogenic inputs, it is common for the spatial distributions of TMs in soils to be spatially correlated. Therefore, the use of combined physicochemical and geostatistical methods for studying the spatial variability of TMs in soils can provide basic information to identify possible sources of contamination (Webster et al., 1994) and the environmental risks (Santos-Francés et al., 2017).

Studies on the heavy metal content of soils in the TNP area have been conducted by numerous teams, focusing on different aspects and on different areas of TNP. Kubica et al., (2002, 2007) investigated the concentrations of selected radionuclides and heavy metals (Zn, Pb, Cd, Ni, and Fe) in soil samples from the Kościeliska, Rybi Potok and Chochołowska Valleys. Wieczorek and Zadrożny (2013) and Kowalska et al., (2021) conducted analyses of the TM content of podzolic soils in selected areas of TNP, linking the content to the podzolisation process. Miechówka and Niemyska-Łukaszuk (2004) studied the Zn, Pb and Cd content in Lithic Leptosols of the TNP area, finding that the diversity of these metal contents was determined by the parent material and diverse soil properties, such as pH and organic-matter content. The total content of Cd, Cr, Co, Mn, Ni, Pb, and Zn in soils belonging to different taxonomical units from the area of TNP were established by Miechówka et al., (2002). Kwapuliński et al., (2013) discussed the speciation of heavy metal forms with reference to the species composition of tree stands in selected areas of TNP, while Paukszto and Mirosławski (2019) studied the relationship between heavy-metal content in soils and nettles (*Urtica dioica*). Soil altitude has been indicated as an important factor that influences the absorption of air-borne pollutants and their concentrations in mountain soils by various authors (Szopka et al., 2013; Tomaškin et al., 2013; Yang, 2021). Korzeniowska and Krąż (2020) studied the differences in heavy metal content in soils from around Morskie Oko Lake and the environs of Kasprowy Wierch Mountain in TNP with increasing altitude, while Miechówka and Niemyska-Łukaszuk (2004) looked for relationships between heavy metal content and altitude in Lithic Leptosols. Similar studies have been conducted in other protected areas in the Polish mountains, such as Karkonosze National Park in the Sudeten Mountains (Szopka et al., 2013) and Babiogórski National Park (Łyszczarz et al., 2020).

To the best of our knowledge, no study has yet been conducted on the spatial distribution of Zn, Pb and Cd in the non-forest part of TNP, nor on their influencing factors, nor on the risks they pose. Determining such information would serve to both records the regional environmental-quality threat and provide information on the health of the ecosystem. For these reasons, our main aim was to establish the main driving factors behind the accumulation and possible sources of Zn, Pb and Cd. The realisation of this aim was achieved through: (i) determination of the Zn, Pb and Zn content in the uppermost horizons and parent material of soils sampled from the area of TNP, which were derived from carbonate-bearing and carbonate-free rocks. The soils differed in their location parameters, including exposure, slope and altitude; (ii) investigation of the spatial distributions of these metals; and (iii) examination of the risks linked to these metal accumulations.

We hypothesised that, after the parent rock, the geographic factors (vegetation belt, slope and exposure) would be the most important in controlling the distribution of Zn, Pb and Cd in the uppermost layers of the non-forest soils.

## Natural characteristics of the study area

**Fig. 1:**
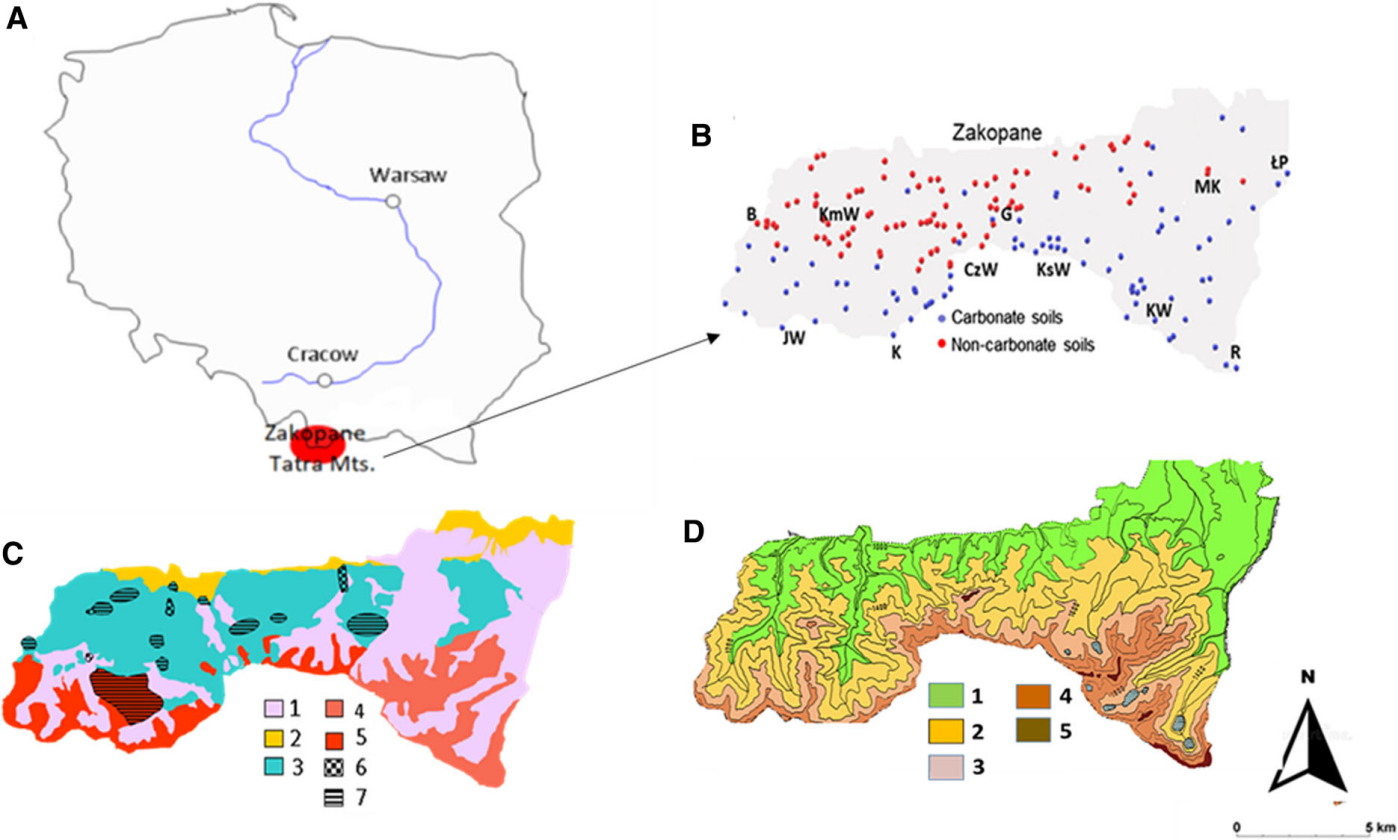
Maps of the Tatra Mountains in Poland, A- Location of the Tatra Mts. on the map of Poland, B- sampling sites on the area of the Tatra National Park, divided into carbonate-containing soils and carbonate-free soils, abbreviations stand for names of mountane peaks: B—Bobrowiec, KmW—Kominiarski Wierch, JW—Jarzabczy Wierch, K—Kamienista, CzW—Czerwone Wierchy, G—Giewont, KsW—Kasprowy Wierch, KW—Kozi Wierch, MK—Mala Koszysta, R—Rysy and a glade: ŁP—Lysa Polana, C—Geological map of the Tatra Mountains with marked remains of the former mining and metallurgy. 1—fluvial and fluvioglacial sediments, slope landslides (Quaternary), 2—Podhale Flysch, Eocene limestones (Tertiary), 3—Carbonate rocks and other sedimentary rocks (Mesozoic), 4—granites of the High Tatra Mts. (Paleozoic), 5—metamorphic rocks and granites of the Western Tatra Mts (Paleozoic), 6—centers of metal works, 7—areas of ore exploitation (after Gorecki & Sermet, 2012), D—Distribution of vegetation belts in the Polish Tatras. 1—lower montane belt, 2—upper montane belt, 3—subalpine belt, 4—alpine belt, 5—subnival belt (after Piękoś-Mirkowa et al., 1996)

The Tatra Mountains are the highest range in the Carpathian Mountains, which spread over a distance of about 1300 km, passing through several Central and Eastern European countries. The Tatra Mountains are characterised by an alpine relief, formed during the Pleistocene glaciations (Klimaszewski, 1988). The Polish part of the Tatras constitutes about one-fifth of the entire range, located in the southern part of Poland, and is preserved as the TNP (Fig. 1A). The southern part of TNP comprises the crystalline massif built of Palaeozoic granites, granodiorites, metamorphic shales, and gneisses, while the northern and central parts are built from Mesozoic sedimentary rocks, such as quartzite, dolomite, limestone, marls, shales, and sandstones (Fig. 1B). In the area of TNP, Cenozoic rocks, such as Eocene limestones and the Podhale Flysch, and Quaternary glacial deposits occur (Piotrowska et al., 2015).

The mean annual temperature ranges from – 4 °C on the northern slopes of the highest peaks of the Tatra ridge to about 6 °C at elevations of 600–650 m at the bottom of the Nowy Targ Basin. The winds are predominantly southerly on the northern side and westerly at the base of the Tatras (in the Orawa–Nowy Targ Basin). The mean annual precipitation exceeds 1500 mm (Niedźwiedź 2015). The temperature decreases and rainfall increases with altitude (Żmudzka et al., 2015).

The Tatras host lower montane, upper montane, subalpine, alpine, and subnival vegetation belts (Fig. 1 C). The montane belts (up to 1550 m a.s.l.) are dominated by forest communities of *Plagiothecio–Piceetum*, *Polysticho–Piceetum* and *Dentario glandulosae–Fagetum*. Non-forest communities represented in the glades and on carbonate rocks mainly by semi-natural vegetation comprising moist or slightly moist hay meadows (*Gladiolo–Agrostietum, Cirsietum rivularis*) and poor acid grasslands (*Polygalo–Nardetum*, *Geo montani–Nardetum*), while in the montane belts, rocky grasslands (mainly *Carici–Festucetum tatrae*) occur. Above the upper timberline, the subalpine belt extends from 1550 to 1800 m a.s.l. and is occupied by the *Pinetum mughi carpaticum* association. High mountain grasslands dominate in the alpine belt, at altitudes of 1800–2300 m a.s.l. The two most important grassland associations that are widely distributed in the Tatras are *Oreochloo distichae–Juncetum trifidi* (on soils derived from carbonate-free rocks) and *Festuco versicoloris–Seslerietum tatrae* (on soils derived from carbonate-bearing rocks). In the subnival belt (above 2250 m a.s.l.), the main community is the *Oreochloetum distichae subnivale* association (Mirek & Piękoś-Mirkowa, 1992).

Geological diversity and variability of climatic conditions and vegetation along with the altitude determine the great diversity of soils in the Tatra Mountains (Miechówka & Drewnik, 2018; Miechówka et al., 2021). In the non-carbonate soils we studied, the share of soils belonging to different main units according to WRB (2015) was as follows: Leptosols (36%), Umbrisols (28%), Podzols (16%), Cambisols (11%), Regosols (7%) and Histosols, and Gleysols—1% each. In the group of carbonate soils, Leptosols (38%) were also the most numerous, followed by Cambisols (33%) and Phaeozems (21%). In addition, this group included Histosols (5%), Gleysols (2%) and Stagnosols (1%). Thus, the Leptosols (65%) dominated among all the studied soils, of which 2/3 were Lithic Leptosols.

## Sampling

For the study soil and parent rock, samples were collected. Soil samples were taken from soil surface horizons, while parent rock samples were taken from the bottom of each pit, in non-forest areas of TNP. The TNP is a compact area, extending between 19^o^45′36″ and 20^o^08′00″E and 49^o^10′42″ and 49^o^20′05″N. The maximum extent of this area in a straight line from east to west is 27.1, and 12 km from north to south. The entire area of TNP is about 212 km^2^, of which 36.3% (about 77 km^2^) is non-forested, covered with vegetation representing natural or semi-natural communities. The study sites were selected in order to have samples representing different locations (slope, altitude and exposition), and a similar amount of soil samples derived from rocks containing carbonates (95) – referred later to as carbonate soils and carbonate-free rocks (82) referred to as non-carbonate soils, basing on the information taken from the geological map. Soil and parent rock samples were taken from 177 non-forest sites, averaging one site per 0.4 km^2^ (Fig. 1A).

The samples were taken at different altitudes (926–2365 m a.s.l.) in such a way that the soils of all the well-developed climatic vegetation belts in the Tatras were represented (i.e. lower montane belt––44 samples, upper montane belt––32, subalpine belt––57, alpine and subnival belts––44).

In the alpine and subalpine belts, the samples were taken based on the course of the main ridge and certain side ridges on either side (so that slopes with different exposures were represented), and on both sides and on the bottom of valleys on the slopes with different exposures, at more or less every 200 m of altitude. In the montane belts, the sampling covered glades, non-forested screes and gullies, and montane calcareous rocks (denuded rock outliers). Soil samples were collected from small glades at one site, and from large glades at several sites (in plant patches representing different plant communities), providing 50 samples in total. The remaining soil samples were taken from montane calcareous rocks (13) and gullies and screes (13).

## Methods

### Laboratory analyses

Analyses were performed on air-dried, 2-mm-sieved soil and ground parent rocks. Measurements of the potentiometric pH were taken using a standard combination electrode and a CPI-551 Elmetron pH meter in distilled H_2_O at a ratio of 1 (soil):2.5 (water) (Tan, 2005). The soil organic carbon content (C) was determined by the modified Walkley–Black wet-combustion method (using external heating), using 0.1 M K_2_Cr_2_O_7_ solution with the addition of concentrated H_2_SO_4_ (Tan, 2005). The sum of the exchangeable base cations (S) was measured after extraction of the individual cations (Ca^2+^, Mg^2+^, K^+^, Na^+^) using 1 M NH_4_Cl at pH 8.2 and the inductively coupled plasma–optical emission spectrometry (ICP–OES) technique. The hydrolytic acidity (HA) was determined through treatment with 1 M Ca(CH_3_COO)_2_ using a 1:2.5 soil:solution ratio. The suspensions were shaken for 1 h, then filtered, and titrated using 0.1 M NaOH to pH 8.2. The total acidity was calculated from the amount of base used (Ostrowska et al., 1991). The soil carbonate CO_2_ (CO_2_) was determined using the volumetric calcimeter method (Food and Agriculture Organization 2020).

The total Zn, Pb and Cd content in the soil and parent rock samples (Zn R, Pb R, Cd R) were determined by digestion in HNO_3_ and HClO_4_ (Hendershof et al., 2006), then measurement using a PerkinElmer atomic emission spectrometer (ICP–OES Optima 7300 DV) and multi-element ICP-IV Merck standard solution. The accuracy of the analytical methods was verified using GSS-8-certified reference material (GBW 07,408, State Bureau of Metrology, Beijing, China), while the precision of the method for determining HMs in the soils was controlled as follows:

1 $${\text{Precision }} = \frac{{\text{Standard deviation}}}{{\text{Mean element content}}} \times { 1}00\%$$

Parameters of the validation method of TMs determination are presented in Table 1S (supplementary material).

**Table 1:** Descriptive statistics of basic properties of soil top horizons and Zn, Pb, Cd contents in soils and parent rocks

Parameter	Mean	Median	Minimum	Maximum	SD	CV (%)	Target -intervention values^*^
*Non-carbonate soils, N* = *82*							
pH	3.5^a**^	3.4	2.4	5.5	0.55	15.6	
C	11.46^a^	9.09	3.01	34.09	6.76	59.0	
S	2.69^a^	1.38	0.32	19.75	3.35	124.7	
HA	29.70^b^	27.69	4.42	100.54	17.43	58.68	
Zn R	55.64^a^	46.35	4.09	358.77	46.73	84.0	
Pb R	23.92^a^	20.76	2.99	134.33	17.98	75.2	
Cd R	0.23^a^	0.15	0.01	0.90	0.23	101.1	
Zn	71.21^a^	59.40	17.90	265.45	46.22	64.91	140–720
Pb	85.82^a^	78.50	21.71	296.76	43.80	51.0	85–530
Cd	1.12^a^	0.84	0.01	8.00	1.08	96.5	0.8–12
*Carbonate soils, N* = *95*							
pH	5.7^b^	6.1	3.9	7.6	1.27	22.3	
C	12.46^a^	8.85	1.44	50.98	9.58	76.9	
S	25.87^b^	23.75	0.98	88.10	18.96	73.3	
HA	8.14^a^	3.80	0.47	42.47	9.12	112.0	
CO_2_	4.16	0.48	0.00	35.28	7.86	188.7	
Zn R	84.21^b^	57.25	5.10	667.65	94.76	112.5	
Pb R	53.03^b^	37.70	3.07	373.69	51.40	97.0	
Cd R	0.73^b^	0.36	0.01	6.03	1.12	152.6	
Zn	226.82^b^	183.13	80.69	688.45	122.79	54.1	140–720
Pb	114.74^b^	89.79	37.85	365.18	70.75	61.7	85–530
Cd	3.36^b^	2.72	0.40	16.31	2.58	76.8	0.8–12

*Dutch standard values for soil (VROM 2013),

**Different letters indicate significant differences at *p* < 0.05,

*C* % organic carbon, *S* Sum of basic cations (Na^+^, Ca^2+^, Mg^2+^, K^+^) in _c_mol^+^/kg, *HA* Hydrolytic acidity in _c_mol^+^/kg, CO_2_ -carbonate CO_2_ in %, Zn, Pb, Cd contents in soils and Zn R_,_Pb R, Cd R contents in rocks in mg/kg

### Statistical and geostatistical analyses

The descriptive statistics (mean, median, maximum, minimum, standard deviation, variability coefficient) of the basic soil properties were calculated. Pearson’s correlation matrix for the quantitative data and Spearman’s rank correlation for the qualitative data were calculated. The post hoc Bonferroni correction (at *p* < 0*.*05) was employed to estimate the least significant differences between the mean values of homogenous groups. PCA was applied to identify the importance of the potential factors affecting the Zn, Pb and Cd content. PCA provides the percentage of variation explained by a given soil property while eliminating the strongly correlated ones. It uses an orthogonal transformation to convert a set of observations of possibly correlated variables into a set of values of linearly uncorrelated variables called principal components. This transformation is defined in such a way that the first principal component has the largest possible variance, and each succeeding component, in turn, has the highest variance possible under the constraint that it is orthogonal to the preceding components (Kukier et al., 2009). Within the main components, variables with a high load factor were considered. A high load factor was defined as an absolute value within 10% of the highest load factor value (Andrews et al., 2002). Two-factor analysis of variance was used to examine the relationship between the TM content and a given factor in the carbonate and non-carbonate soils. In order to meet the principles of the analysis of variance (additivity, homogeneity of variance, and normality of distribution), the data were subjected to logarithmic transformation prior to the analysis. All statistical analyses were conducted using Statistica PL v. 13 software (StatSoft Inc., 2014, Poland).

In order to describe the organisation and regularity of the TMs in the space considered, semivariograms were created. These estimated the semivariance, *γ*(*h*), of a variable measured at two points from *h*. Semivariograms show how the information between the two measured points of a variable degrades as the distance increases. The semivariograms were then empirically fitted by a mathematical function comprising a sill. The fitting enabled information on the spatial structure of the variable to be determined using the following parameters: the nugget (the value of the y-intercept, representing the part of the variability lower than the sampling interval), the sill, and the range (the distance from which the sill is reached and beyond which there is no longer any autocorrelation). The ratio between the nugget and the total variance (the nugget:variance ratio) was calculated in order to have a relative assessment of the nugget effect (expressed as a percentage). Then, maps of the spatial distributions of the Zn, Pb and Cd as well as slope steepness in the studied soils were created using the kriging method for spatial interpolation. Both the semivariograms and the maps were created using Surfer 20.0 software.

### Calculation of soil pollution indices

The Nemerov Pollution Index (PI_Nemerov_) assesses the degree of contamination in a soil environment (Ogunkunle & Fatoba, 2013; Qing et al., 2015).

2 $$PI_{{{\text{Nemerov}}}} = \sqrt {\frac{{\left( {\frac{1}{m}\mathop \sum \nolimits_{i = 1}^{m} Pi} \right)^{2} + Pi^{2} \max }}{m}}$$

where *Pi* is the pollution index of a particular heavy metal, calculated as *Pi* = $\frac{C}{B}$, where *C* is the heavy metal content determined in the uppermost soil horizon and *B* is the heavy metal content of the parent material (geochemical background), *Pi*_max_ is the maximum value of the pollution index of all heavy metals, and *m* is the number of heavy metals studied.

The pollution categories based on *PI*_Nemerov_, as established by Zhong et al., (2010), are ≤ 0.7––excellent, 0.7–1––clean, 1–2––slight pollution, 2–3––moderate pollution, and ≥ 3––heavy pollution.

The potential ecological risk (PER) indicates the degree of environmental risk caused by the concentration of a heavy metal in soil.

3 $$\mathrm{PER }= {\sum }_{i=1}^{m}{\mathrm{Er}}^{i}$$

4 $${\mathrm{Er}}^{i} ={Tr}^{i}\mathrm{x }Pi$$

where *Er* is the single ecological risk factor index, *m* is the number of heavy metals studied, *Tr*^*i*^ is the toxicity response coefficient of heavy metals (Håkanson, 1980), and *Pi* is the single heavy metal pollution index. The PER categories, according to Håkanson (1980), are ≤ 90––low, 90–180––moderate, 180–360––strong, 360–720––very strong, and ≥ 720––immensely strong.

## Results

### Spatial distribution analysis

**Fig. 2:**
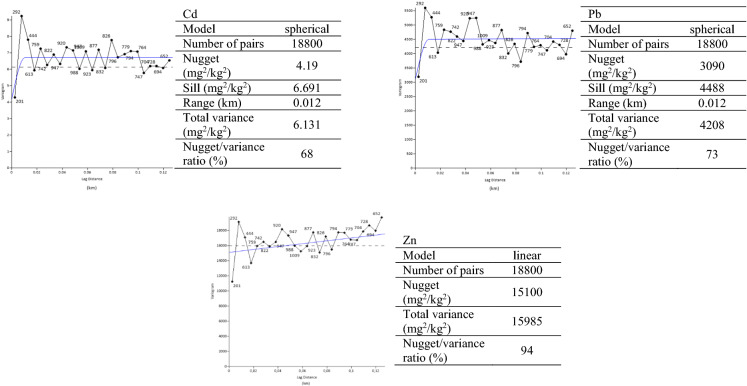
Omnidirectional semivariograms, experimental points and fitted with spherical models for Cd and Pb, and linear for Zn. The dashed line corresponds to the total variance. The data at the right of each graph describe the modelled semivariograms

A spatial dimension of the Zn, Pb and Cd concentrations in the uppermost layers of non-forest TNP soils is presented in their calculated semivariograms, shown in Fig. 2, together with the parameters (nugget effect, range and nugget:variance ratio) shown to the right of each variogram. The properties of the Pb and Cd semivariograms showed a high degree of similarity, with small ranges and a clearly marked nugget effects. The nugget effects resulted both from measurement errors and spatial sources of variation at distances smaller than the sampling intervals. The shapes of the variograms, and the nugget:variance ratios of 68 and 73%, respectively, for Cd and Pb showed a moderate spatial dependence of these metal concentrations. The Zn variogram was dominated by the nugget effect, with a nugget:variance ratio of 94%, indicating a lack of spatial continuity of Zn content in the soil samples.

**Fig. 3:**
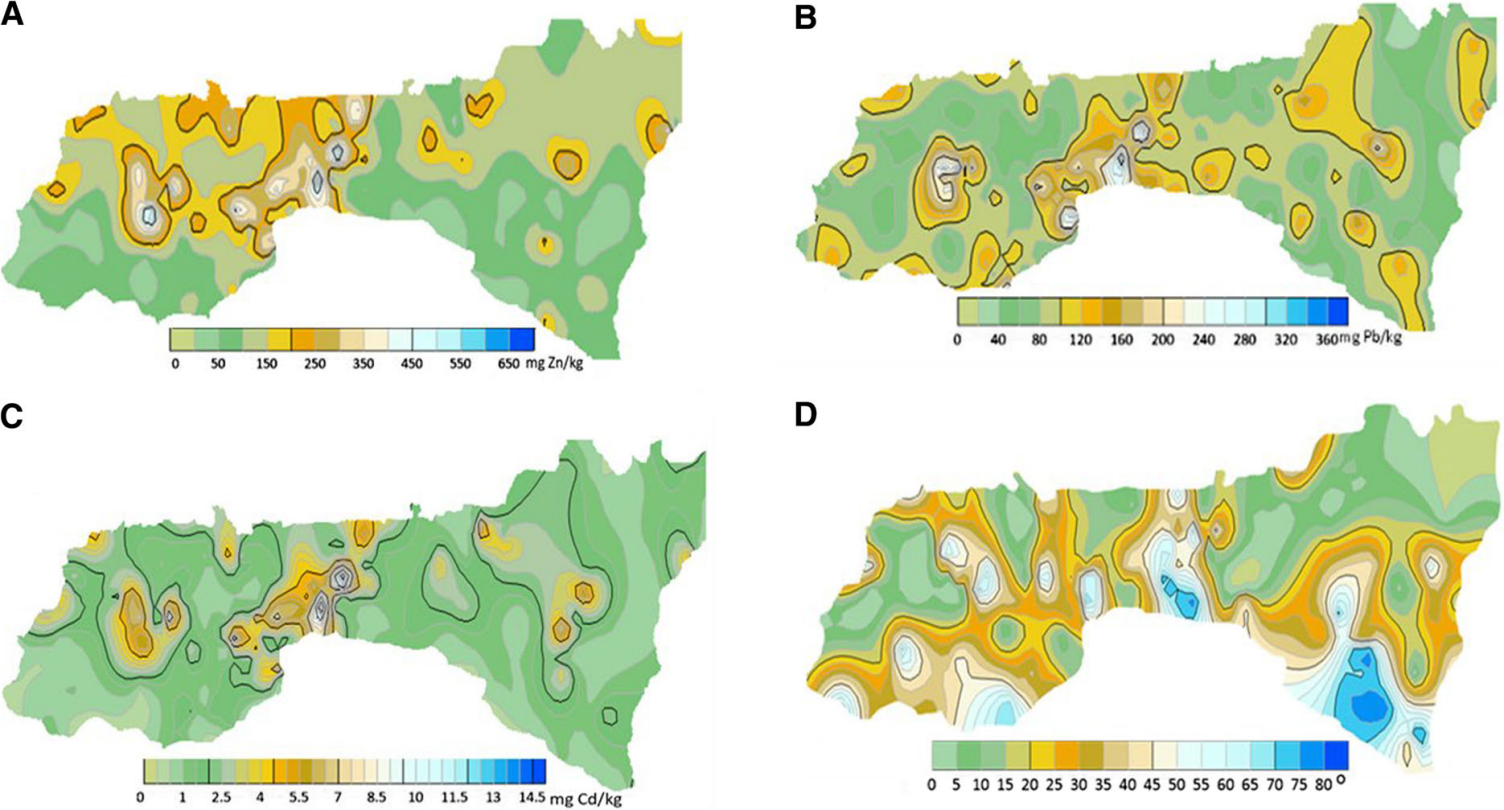
**A**, **B**, **C** Spatial distribution of Zn, Pb, Cd, and D- slope steepness as results of a kriing method

The distribution of Zn, Pb and Cd is presented in map form in Fig. 3 A–C. All three metals had a few areas of strong accumulation, such as between the Czerwone Wierchy (CzW), Giewont (G) and Kasprowy Wierch (KsW) mountain peaks towards Zakopane, as well as around the peak of Kominiarski Wierch (KmW). Higher amounts of Cd were also determined around Mala Koszysta (MK), while Zn was more dispersed, having areas of accumulation in the western part of TNP, especially towards its northern border. Comparing the areas of Zn and Cd accumulation (Fig. 3 A, C) with the sampling points on the map in Fig. 1A, it can be seen that the areas of higher accumulation of these metals occurred in soils derived from carbonate-containing rocks. Conversely, Pb, apart from the areas of common accumulation with the other two TMs, occurred in high amounts in a few compact areas distributed all over TNP.

### Basic soil characteristics

The results of the laboratory analyses indicated that lower mean values of the basic properties (pH, S), as well as the Zn, Pb and Cd content, occurred in the non-carbonate rather than carbonate soil and parent rock samples (Table 1). Only higher mean HA values were determined in the non-carbonate soils, while the C content did not differ significantly between the soils of the two groups. The mean content of the three TMs in the carbonate soils exceeded the Dutch standard target values (Ministerie van Volkshuisvesting Ruimtelijke Ordening Milieubeheer, 2013). In the non-carbonate soils, these target values were exceeded only by the mean Pb and Cd content. However, the maximal Zn values in these soils also exceeded the target values. The maximal Cd content in the carbonate soils even exceeded the intervention value.

### First approach to the origin of the Zn, Pb and Cd in the soils based on principle component analysis

#### Non-carbonate soils

The first principal component (PC1), which explained 22.8% of the Zn variance, was determined by HA (25% of the variance), C (24%) and Pb (12%). PC2 explained 21.1% of the Zn variance and was determined by vegetation belt, slope and exposition, which explained 22, 18 and 16% of the variance, respectively (Fig. 3). A third PC (15.5% of the Zn variance) comprised mainly the Zn and Pb content in the parent rock (27 and 30%, respectively, positively correlated), and a fourth (12.0%) comprised pH and S, each explaining 19% of the Zn variance. These first four PCs, with a Kaiser value > 1, together explained 71.5% of the Zn variance.

PC1, which explained 21.7% of the Pb variance, was affected mainly by the slope (24% of the variance), vegetation belt (20%), and exposure (19%), while PC2 explained 21.2% of the Pb variance and was determined by pH (30%) and HA (26%) (Fig. 3). PC3, which explained 15% of the Pb variance, was dominated by the Zn and Pb content of the parent rocks (43 and 33% of the variance, respectively), whilst PC4 (14.3% of the variance) was determined by the C, Zn and Cd content of the soils (22, 17 and 19% of the variance, respectively). Altogether, PC1–4 explained 72.2% of the Pb variance.

The Cd variance explained by PC1 (22.7%) was affected mainly by HA (29% of the variance), C (26%) and pH (17%). PC2 explained 21.1% of the Cd variance and was determined mainly by vegetation belt (22% of the variance), slope (21%) and exposure (17%) (Fig. 3). PC3 (16.6%) was explained by the soil Zn and Pb content (respectively, 34 and 19%), while PC4 (14.7%) was determined by the parent-rock Zn and Pb content (39 and 36%, respectively). Altogether, PC1–4 explained 75% of the Cd variance.

#### Carbonate soils

**Fig. 4:**
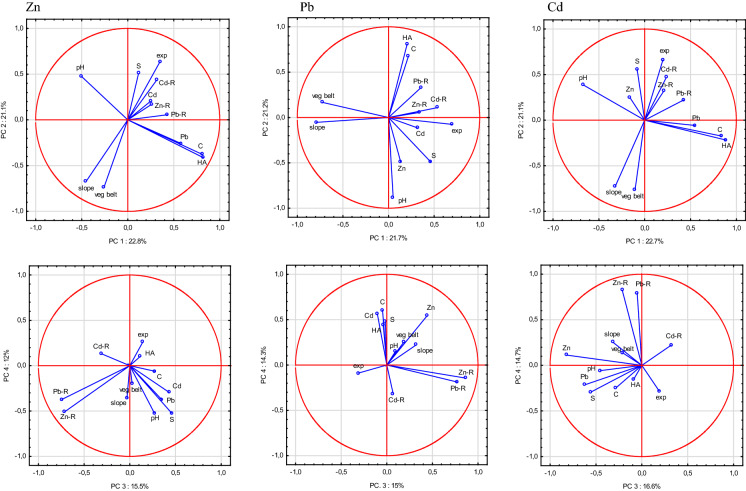
PCA model for factor importance for Zn, Pb and Cd in non-carbonate soils

The Zn variance was mainly explained (61.9%) by three PCs, which had a Kaiser value > 1. PC1 explained 31.0% of the variance and was affected by the soil Pb and Cd content (16 and 15% of the variance, respectively), as well as the parent-rock Zn and Pb (12 and 17%, respectively). PC2, explaining 19.1% of the Zn variance, was related to pH (34% of the variance), HA (25%), CO_2_ (15%) and S (14%), while PC3 (11.8% of the Zn variance) was connected with exposure (29%), slope (24%) and vegetation belt (17%) (Fig. 4).

Similar to the Zn variance, PC1, explaining 31.2% of the Pb variance, was mainly affected by the soil Zn and Cd content (both 16%) and the parent-rock Zn, Pb and Cd content (13, 17 and 13%, respectively) (Fig. 4). PC2 (19.2% of the Pb variance) was explained mainly by pH (34% of the variance), HA (26%), CO_2_ (14%), and S (13%), while PC3 (11.7%) was affected by exposure (31%), slope (28%) and vegetation belt (17%). These three factors together explained 62.0% of the Pb variance.

The Cd variance in the carbonate soils was explained (61.4%) by the first three PCs, with a Kaiser value > 1. PC1 explained 31.3% of the Cd variance. As for Zn and Pb, PC1 was determined mainly by the parent-rock Pb, Zn and Cd content (14, 17 and 11%, respectively) and the soil Zn (16%) and Pb (15%) content (Fig. 4). PC2, which explained 18.4% of the Cd variance, was determined mainly by pH (35% of the variance), followed by HA (25%), S (15%) and CO_2_ (14%), while PC3 (11.7%) was affected mainly by exposure (29%), slope (27%) and vegetation belt (18%).

About 50% of the variance of Zn, Pb and Cd in the carbonate soils was explained by the first two principal components, which is more than in the non-carbonate soils (about 43%) (Figs. 3, 4).

### Heavy metal contamination indices

**Fig. 5:**
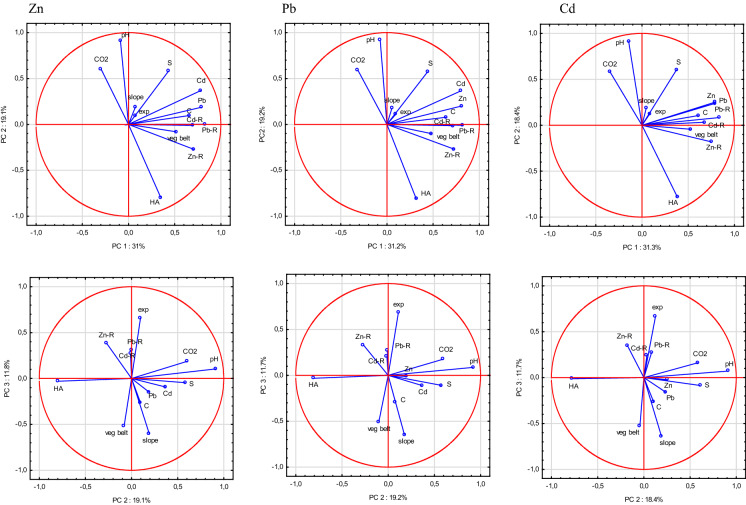
PCA model for factor importance for Zn, Pb and Cd in carbonate-containing soils

The *PI*_Nemerov_ values calculated for the carbonate and non-carbonate soils indicated heavy pollution for about 90% and moderate pollution for about 10% of these in both groups (Fig. 5A). Based on the PER values, the soil ecological risk was immensely strong for 30% of the non-carbonate and 19% of the carbonate soils, very strong for about 12% of the soils in both groups, strong for 20% of the carbonate soils and less than 10% of the non-carbonate soils, and moderate and low for about 20% of the soils in both groups (Fig. 5B).

## Discussion

### Possible trace-metal sources related to their spatial distributions

In mountainous areas, the TM distribution has been found to be highly variable and spotty (Hajdúk, 1988; Szopka et al., 2013). Consequently, prediction of their spatial distributions using simple geostatistical models is quite difficult (Szopka et al., 2013). The high nugget effects and small sills observed in the TM variograms, and the moderate spatial correlation between Pb and Cd and the lack of such a correlation with Zn, confirm this opinion. According to Tóth et al., (2016), a short-range variability in TMs is considered to be a regional scale TMs distribution, arising from local mining or industrial activities, or from a diversity of geological formations. Therefore, the low spatial correlation between Pb and Cd may indicate the deposition of atmospheric dust-containing metals emitted from local road transport, especially in the vicinity of parking lots. Although soil samples were not taken from locations that had been most exposed to this type of pollution, it is not possible to eliminate the influence of vehicular traffic on the heightened TM content in the soils from Łysa Polana and Polana Huciska (in the Chochołowska Valley). In the ‘80 s of the previous century, the yearly precipitation of atmospheric dust on the area of TNP amounted to about 27 t/km^2^, including 40.1 and 5.16 Cd/km^2^/year. Results of the mineralogical composition of the dusts indicated the prevalence of particles of anthropogenic origin such as glass and heavy metal balls, coke breezes, and iron oxide grains (Schejbal-Chwastek & Tarkowski, 1988). Moreover, the Pb and Cd distribution may also have been transported long distances as metals and/or from ski-lift machinery, the maintenance of which could potentially have introduced petroleum products into the area in the form of TM-containing solvents, grease, or gasoline (Ciarkowska, 2018; Walter, 2001). As TNP directly borders with Zakopane, emission from Zakopane strongly affects the natural environment of the TNP area. According to the data provided by the Statistics Office, in Zakopane heavy metals are emitted with dust, exceeding the permissible level of particulate PM10 in the air (about 120% of permissible value) in a calendar year (Ciećko, 2016). Results of Schejbal-Chwastek and Tarkowski (1988) indicated also that big power plants with high chimneys were the main emitters, thus about 70% of falling dust of the area of TNP was probably of a long-range origin, mainly from Krakow and Silesia areas which are located at the distance of 100–150 km from the Tatras. In eastern Krakow, a large industrial centre, which reached maximum production in the 1970s and ‘80 s, emitted dust and gaseous pollutants, including those containing TMs, amounting to more than 100,000 tonnes into the atmosphere annually, and similar amounts were emitted from the power plants in the Silesia region (Ciarkowska & Gambus, 2020, Central Statistics Office 2020). Although in the twenty-first century amounts of dust produced by plants in Krakow and Silesia region decreased several times, the trace metals deposited remained accumulated in the soil.

The lack of spatial correlation with Zn may be related to its increased presence in dispersed historical mine sites, where ores were also processed. Human interference in the natural environment of the Tatra Mountains has been taking place since medieval times, at different spatial and temporal intensities, as is apparent from various historical materials (Rączkowska, 2019). When taking the soil samples, efforts were made to avoid such sites, but the high Zn content in the soils from glades where iron ores were processed or transported through (Wyżnia Kira Mietusia––174 mg kg^−1^, Polana Smytnia––295 mg kg^−1^, Glade Dudowa––326, 466 mg kg^−1^, Huty Lejowe––167 mg kg^−1^) may have resulted from such activities. The Zn content in the soils may also have come from the parent rocks, ore-bearing rocks being randomly distributed in the environs of TNP (Rączkowska, 2019).

### Main factors affecting trace-metal accumulation

#### Parent rocks

Several authors have confirmed that the parent rock, especially in mountainous areas, exerts a strong control on TM concentrations and their variability in the soils derived from them (Atteia et al., 1995; Fernández et al., 2018; Miechówka & Niemyska-Łukaszuk, 2004; Utermann et al., 2019). In TNP, the carbonate-bearing and carbonate-free rocks differed significantly in their TM content. The mean amounts of Zn, Pb and Cd were, respectively, 1.5, 2.3 and 3.2 times greater in the rocks containing carbonates than in the carbonate-free rocks. Similarly, Barančoková et al., (2009) found the highest Pb content to occur in the Triassic limestones (Gutenstein Limestone) and shales of the Carpathian Keuper in the Belianske Tatras. Our results accord with the findings of Miechówka & Niemyska-Łukaszuk (2002) who stated that the mean TM content in rocks containing carbonates is higher than in carbonate-free rocks, although this is not always the case. These authors found high concentrations of Zn, Pb and Cd in amphibolites, biotite granodiorites, chalk marls, and Triassic and graphitoid shales. In the limestones and dolomites, they often established significant amounts of Pb and Cd, while high concentrations of Zn occurred in the granodiorites. The rocks from this study with the lowest amounts of TMs were white granites and granite-gneisses, while the sandstones and Triassic quartzites were also very low in Zn and Pb. Thus, the bedrock is often the main factor that dictates the elemental composition of a soil. The results of the PCA confirmed this, indicating that the TM content of the parent rock was an important factor (PC1 in the carbonate soils), or at least a subordinate factor (PC3 in the non-carbonate soils), in the TM distributions. However, other factors, of natural and anthropogenic origin, may be superimposed, causing high variability in the TM concentrations and their distribution in soils, even over small areas (Fernández et al., 2018; Hajdúk, 1988; Krami et al., 2013). For these reasons, in order to identify the other factors affecting the Zn, Pb and Cd accumulations in the uppermost soil horizons, the carbonate and non-carbonate soils were analysed separately.

#### Soil properties

The results of the PCA indicated that the soil chemical properties (pH, C content and HA) were the most important factors affecting the variable concentration of Zn and Cd in the non-carbonate soils, and a factor of secondary importance for Zn, Cd and Pb in the carbonate soils. These properties determine the solubility or binding of TMs, which tend to be leached out of soils, depending on the pH and C content (Chai et al., 2015; Hudec et al., 2013; Quenea et al., 2009). In fact, in the non-carbonate soils, pH was positively correlated with Zn (Pearson correlation coefficient = 0.4649, *p* < 0.05), while the amount of Pb depended strongly on SOC storage (Pearson correlation coefficient = 0.5835, *p* < 0.05), similarly to what has been reported by several authors from other mountain ranges (Kaste et al., 2005; Łyszczarz et al., 2020; Szopka et al., 2013; Wang et al., 2009). In the carbonate soils, Cd was correlated with pH (0.2539) and all three metals were correlated with SOC content (0.4356 for Zn, 0.4925 for Pb, and 0.5960 for Cd, *p* < 0.05). These relationships may indicate that non-forest meadow soils, especially in montane areas, act as sinks for heavy metals, their accumulation stimulated by the high amounts of organic matter that are usually present in soils in such locations (Ciarkowska, 2018; Józefowska et al., 2014; Miechówka et al., 2002; Tomaškin et al., 2013, Yang, 2021).

#### Vegetation belt, slope and exposure

**Fig. 6:**
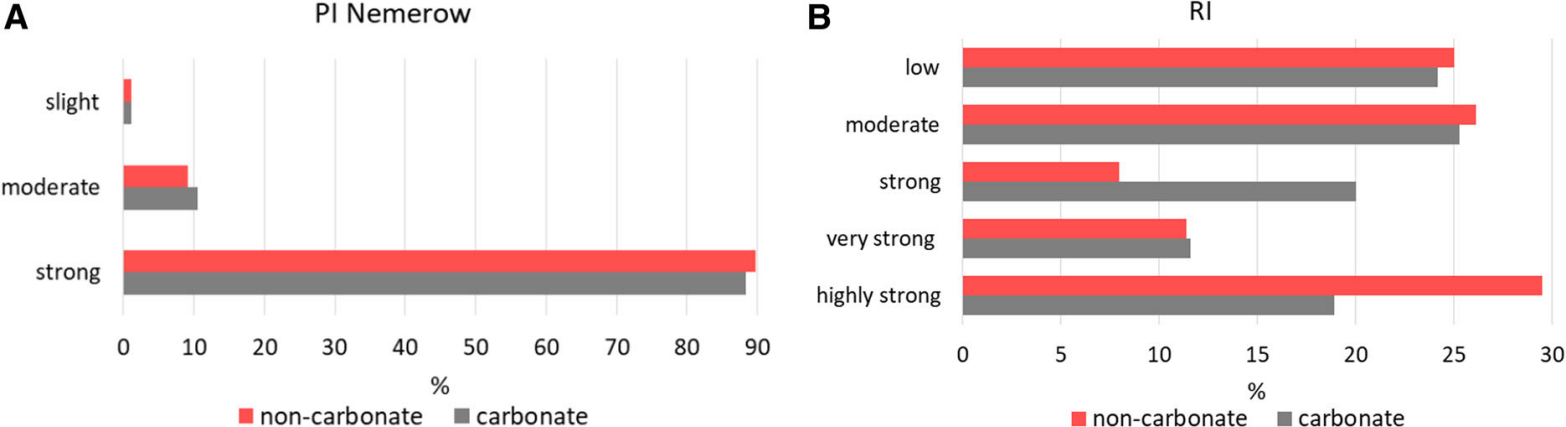
Trace metal contamination indices: **A** share of soils with a given degree of pollution according to PI Nemerow, **B** share of soils posing a given ecological risk (RI)

**Fig. 7:**
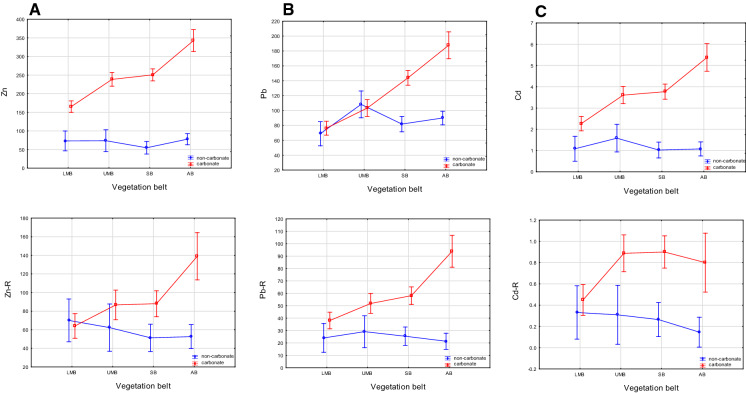
Relationships between trace metal contents in soil (upper graphs) and in parent rock (lower graphs) and a vegetation belt (altitude a.s.l.) in carbonate and non-carbonate soils, **A** Zn, **B** Pb, and **C** Cd. Results of two-factor analysis: soil (carbonate or non-carbonate) and a vegetation belt explaining the accumulation of a given TM. LMB stands for lower montane belt, UMB stands for upper montane belt, SB stands for subalpine belt and AB stands for alpine belt

**Fig. 8:**
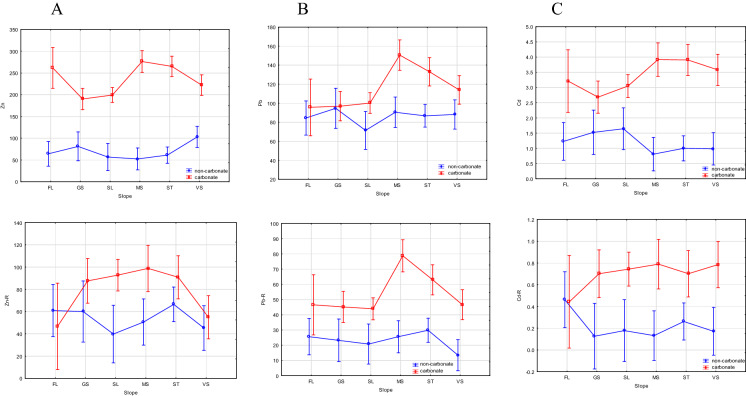
Relationships between trace metal contents in soil (upper graphs) and in parent rock (lower graphs) and a slope steepness in carbonate and non-carbonate soil, **A** Zn, **B ** Pb, and **C** Cd. Results of two-factor analysis: soil (carbonate or non-carbonate) and a slope steepness explaining the accumulation of a given TM. FL stand for flat areas (0–1% slope), GS –gently sloping (2–5% slope), SL- sloping (6–15% slope), MS- moderately steep (16–30% slope), ST- steep (31–60% slope), and VS- very steep (> 60% slope)

According to the PCA, the location effects of exposure, vegetation belt and slope steepness constituted a secondary factor in the non-carbonate soils for Zn and Cd, and the primary factor for Pb, and the tertiary factor in the carbonate soils. To better understand how the TM content differed between the soils and these factors, a two-factor analysis was performed, with the Zn, Pb and Cd content explained by vegetation belt, slope or exposure as the first factor, and the presence of carbonate (in the carbonate and non-carbonate soils) treated as the second factor. This analysis was performed on the soil and parent-rock samples to determine differences in the TM distribution patterns (Figs. 6, 7 and 8). In the carbonate soils, the Zn and Pb content increased with altitude, from the lower montane (LMB) to alpine belts (AB), while in the non-carbonate rocks, Zn was relatively stable, while Pb had a peak in the upper montane belt (UMB) (Fig. 6A, B). Site altitude has been indicated by various authors to be an important factor in the absorption of air-borne pollutants and their concentration in mountain soils, with increasing concentrations of TMs at higher elevations having been described by Smidt and Herman (2004) in the Alps and Tomaškin et al., (2013) in Slovakian national parks. Łyszczarz et al., (2020) observed a similar dependence in the case of Pb in the organic horizons of forest soils, but a reverse dependence for Cd and Zn. However, according to him, the heavy metal content in soil mineral horizons has always been negatively correlated with altitude. Research on the Slovakian Tatras has also shown that the Pb content in mosses increases with increasing altitude (Šoltés, 1992), whereas, in the Polish Tatras, Korzeniowska and Krąż (2020) found decreasing TM content with increasing altitude. Miechówka and Niemyska-Łukaszuk (2004), in a study on Lithic Leptosols, determined an increase in TM content in soils derived from carbonate rocks and a decrease in soils derived from igneous rocks. Bacardit and Camarero (2010), examining the TM content in snow, found the highest TM accumulations at low altitudes (in the Central Pyrenees), whilst Gerdol and Bragazza (2006) recorded the highest TM accumulations in Alpine mosses at moderate elevations.

Other authors, however, including Mutsch, (1996) in the Alps and Szopka et al., (2013) in the Karkonosze Mountains, found no relationship between altitude and metal (particularly Pb) accumulation in soils. Our results showed a similar pattern between Zn and Pb distribution and altitude in the soils as in the parent rocks (Fig. 6A, B), thus strongly suggesting that the soil TM content primarily depends on their distribution in the parent material. Only Cd showed a different distribution between the soils and parent rocks, with an increase in its mean concentration at higher altitudes in carbonate soils and in the non-carbonate soils of the upper montane belt (Fig. 6C). As Cd is a metal that spreads as small particles that can be transported over large areas, its deposition is more closely related to the wind speed, which increases above the tree line (Ciriaková, 2009), and the amount of precipitation, which increases with elevation (Barančoková et al., 2009; Magnani et al., 2018; Yang et al., 2021). A small decrease in Cd content with altitude observed in some non-carbonate soils may be explained by its leaching from adjacent acid soils. Similar dependencies have been previously identified in Lithic Leptosols (Niemyska-Łukaszuk & Miechówka 2004).

In the carbonate soils, a pattern of Zn and Cd distribution associated with slope steepness was revealed, with a decrease in these metal accumulations in soils located on gentle (GS) and slightly steep (SL) slopes compared to their amounts in soils on flat land ( FL) and, especially, on moderately steep (MS) and steep (ST) slopes (Fig. 7A, C). As this pattern is different from their distribution in relation to their parent rocks. These accumulations are likely the result of air-borne pollution. In the non-carbonate soils, Zn showed a slight increase with slope steepness, while the amount of Cd was lower in soils located on steep slopes. Gentle slopes usually occur at lower altitudes, so the lower amounts of pollutants are also connected with lower altitudes; slope steepness and altitude were positively correlated in both the carbonate and non-carbonate soils, with Spearman correlation coefficients of 0.229 and 0.645, respectively (at *p* < 0.05). A decrease in Cd accumulation in soils on steep slopes may be attributable to its leaching from soils under acidic conditions, as mentioned with respect to high altitude. In both the carbonate and non-carbonate soils, the Pb distribution seems not to have been affected much by the slope, instead of exhibiting similar patterns between the soils and their parent materials (Fig. 7B).

**Fig. 9:**
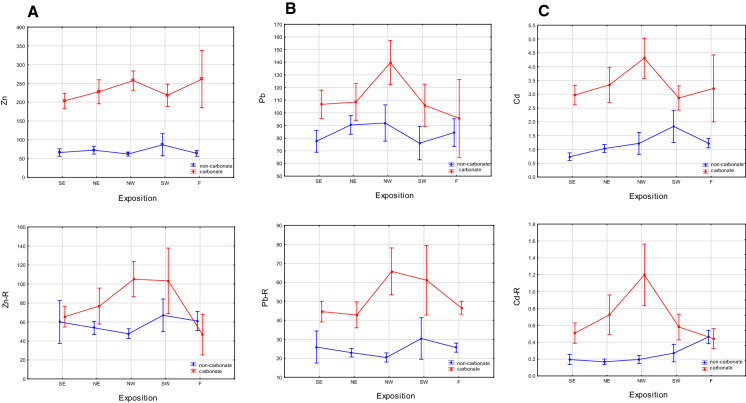
Relationships between trace metal contents in soil (upper graphs) and in parent rock (lower graphs) and an exposition in carbonate and non-carbonate soil, **A** Zn, **B** Pb and **C** Cd. Results of two-factor analysis: soil (carbonate or non-carbonate) and an exposition explaining the accumulation of a given TM. The expositions were grouped: NW included: WNW, NW, NNW, N expositions; NE included: NNE, NE, ENE E expositions; SE included: ESE, SE, SSE, S expositions and SW included: SSW, SW, WSW, W expositions

The deposition of heavy metals is also associated with a site’s exposure to winds blowing from the direction of industrial dust emitters. In the case of the Tatra Mountains, the amount of industrial dust deposition is mainly influenced by northerly and north-westerly winds, which may increase the TM content on the slopes, although local winds (mountain breezes) may weaken this effect (Hess, 1996). In fact, from our results (Fig. 8A–C), the highest accumulations of Zn, Pb and Cd were observed in soils on slopes with north-western exposure, and especially in carbonate soils. Much higher contents of all the TMs were found in the soils on the high massifs, built largely of carbonate rocks, that were collected from protrusions in front of the main ridge of the Tatra Mountains (KW, G, MK) or from the main ridge arc (CzW) (Fig. 1), which means they were exposed to winds bringing pollution from industrial areas such as Silesia, Ostrava and the Krakow region. Northerly and north-westerly winds bringing pollution from these sources located to the west and north, within 150–200 km, were also noted by Barančoková et al., (2009) in the Belianske Tatry Mountains. In the non-carbonate soils, Pb accumulation also occurred on north-west- exposed slopes, while Cd peaked in soils on south-west-facing slopes, not following the pattern of metal distribution in the parent rocks. Thus, these accumulations likely resulted from long-range emissions. With Cd being easily transportable over long distances, it may also have been brought from nearby countries located to the south of Poland, such as the Czech Republic and Slovakia, as emphasised by Paukszto and Mirosławski (2019) in a study of the influence of long-term emissions on metal pollution in the TNP area in soils and stinging nettles. (Fig. 9).

### Ecological risks related to TM pollution in the uppermost layers of TNP soils

The TMs found in the studied non-forest soils of TNP generally exceeded standard limits, but remained below the intervention values. However, several authors have reported unfavourable eco-toxicological effects caused by Pb present in soils at concentrations of 200 mg kg^−1^ (Bååth, 1989; Johansson et al., 2001; Tyler et al., 1989) or even much lower (de Vries et al., 2007). According to Rademacher, (2003), the maximum Pb content tolerable for the soil biota is 70–150 mg kg^−1^ because soil microorganisms and mesofaunas are thought to be much more sensitive indicators of Pb toxicity than plants. The presence of high levels of Zn, Pb and Cd can also have an additive effect on soil faunal components, such as earthworms and collembolans. A critical limit for these organisms was set by de Vries et al., (2007) at 0.9 mg kg^−1^ for Cd and 30 mg kg^−1^ for Pb. Zn concentrations of 243 mg kg^−1^ in soil organic layers and 100 mg kg^−1^ in soil mineral layers are believed to inhibit microbe-mediated ecological processes by 20% (de Vries et al., 2007). Because these amounts were greatly exceeded in the studied soils, it is not surprising that the degree of potential ecological risk from the TMs was indicated as being very or immensely strong (above 70%) in both the carbonate and non-carbonate soils, with a higher risk in the non-carbonate soils. The *PI*_Nemerov_, which assesses the overall level of pollution, was high for almost 90% of the soils. Particularly worrying was an immensely strong risk assessed for the non-carbonate soils, in which TMs remain in a soluble, directly available form due to the acidic soil conditions. High amounts of Zn, Pb and Cd were found by Paukszto and Mirosławski, (2019) in all the morphological parts of stinging nettles, which are often used as good bioindicators of such metals. Concentrations of Pb and Cd in *Vaccinium myrtillus, Luzula luzuloides* and *Dryopteris dilatata* have mostly been found to exceed the limits of the natural occurrence of these elements in plants (Ciriaková, 2009). The alpine biota in the Tatras is probably under permanent, long-term stress from exposure to TMs, as suggested by the excessive amounts of Zn, Pb and Cd reported from the skulls, bones, and teeth of wild animals, such as snow voles, Tatra marmots and chamois (Ballová et al., 2019; Janiga & Haas, 2018; Janiga et al., 2019). However, the pollution of the TNP environment is primarily a sad remnant of the strong development of heavy industry and mining in Poland in the 1970s, with influxes of pollutants also taking place in later years. Even though a recent decrease in Zn and Pb emissions into the air has been recorded, the level of Cd emissions is still on the increase (Central Statistical Office, 2020). For these reasons, studies of the environmental status of mountainous areas, and other areas remote from industrial sources, are necessary in order to increase our awareness of the risks and to enable us to take appropriate legal steps to reduce pollution. Future studies should expand to include trace elements that have not yet been taken into account.

## Conclusions

The amounts of Zn, Pb and Cd accumulated in the surface layers of non-forest soils in the TNP region indicate that a major part of the studied area is heavily polluted and the values of the poses potential ecological risks to varying degrees because these TM concentrations exceed standard limits, with maximal Cd levels even exceeding intervention values at some locations. However, the spatial distribution of the TMs was highly variable, being controlled by different factors. It has already been established that the soil TM content may differ strongly depending on the parent rock composition (here, whether or not it contained carbonates), and we found that the parent rock exerted a primary control on TM distribution. But our results also partially confirmed our hypothesis, which assumed that, besides the bedrock, the most important factor affecting TM distribution would be geographic location. Geographic factors, such as slope, vegetation belt (altitude a.s.l.), and exposure, were found to be of secondary importance in the non-carbonate soils and of tertiary importance in the carbonate soils. In the carbonate soils, the TM content in the parent material was the most important factor controlling their distribution in the soil surface layers. In the non-carbonate soils, the properties influencing TM solubility (pH and SOC content) were more important than the TM content of the parent material. The Zn, Pb and Cd distribution patterns indicated that mainly Cd but also, to a lesser extent, Pb and Zn accumulations resulted from the long-range transport of these TMs from industrialised areas. The Zn concentrations were also strongly affected by local sources, such as historical mining and/or the transport of Zn-bearing ores.

## Supplementary Information

Below is the link to the electronic supplementary material.

Supplementary file1 (DOCX 12 kb)
