# Supplementary material for: Identification of the factors determining the concentration and spatial distribution of Zn, Pb and Cd in the soils of the non-forest Tatra Mountains (southern Poland)
Source: Environ Geochem Health. 2022 Jan 10;44(12):4323–41. doi: 10.1007/s10653-022-01201-3 (PMC9675705; doi:10.1007/s10653-022-01201-3)
Supplement: Supplementary file 1 — Supplementary file1 (DOCX 12 kb) [file 10653_2022_1201_MOESM1_ESM.docx]

Table 1S. Parameters of the validation method of HMs determination

| Element | Content | | Precision | Accuracy | LOD^2^ |
| --- | --- | --- | --- | --- | --- |
|  | Measured | CRM^1^ |  |  |  |
|  | mg kg^-1^ | | % | | µg dm^-1^ |
| Zn | 22.1 ± 0.2 | 20.6 ± 2.2 | 1.31 | 7.00 | 0.0071 |
| Pb | 7.15 ± 0.07 | 7.10 ± 1.10 | 3.22 | 0.70 | 0.0482 |
| Cd | 0.16 ± 0.02 | 0.14 ± 0.06 | 6.78 | 14.3 | 0.0038 |

^1^certified reference material, ^2^limit of detection
